# Gestational Age in Autistic Children and Adolescents: Prevalence and Effects on Autism Phenotype

**DOI:** 10.1007/s10803-022-05466-6

**Published:** 2022-02-07

**Authors:** Miriam I. Martini, Inge Merkelbach, Sander Begeer

**Affiliations:** 1 Department of Medical Epidemiology and Biostatistics, Karolinska Institutet, Nobels Väg 12A, 171 77 Stockholm, Sweden; 2 Erasmus School of Social and Behavioural Sciences Erasmus School of Social and Behavioural Sciences/ Behavioural Change, Burg. Oudlaan 50, Rotterdam, The Netherlands; 3 Department of Clinical, Neuro and Developmental Psychology, Vrije Universiteit Amsterdam, and Amsterdam Public Health Research Institute, Amsterdam, The Netherlands

**Keywords:** Autism spectrum disorders, Preterm birth, Post-term birth, Autism phenotype

## Abstract

Pre- and post-term children show increased autism risk. Little is known about gestational age (GA) prevalence among autistic children, and their respective autism phenotype. We compared prevalence of pre-, full- and post-term birth between a population-derived sample of *N* = 606 (137 females, 22.61%) autistic children and adolescents (mean age = 14.01, SD = 3.63, range 3–24) from the *Netherlands Autism Register*, and matched controls from the Dutch birth register. Autism phenotype and comorbid symptoms were assessed with the AQ-short and SDQ questionnaires. Using logistic regression, we found higher prevalence of pre- and post-term birth among autistic individuals but no phenotypical differences across GA groups. Autism risk was particularly elevated for post-term children, highlighting the need for closer investigation of autism on the whole GA range.

Autism spectrum disorders (ASD, in this paper collectively referred to as autism) are neurodevelopmental disorders associated with difficulties in social interaction and communication, as well as restricted, repetitive patterns of behaviour, interests and activity (APA, 2013). Across the lifespan autism is often accompanied by psychiatric, physical and academic problems, which negatively impact individuals’ well-being and life outcomes including living situation, educational attainment and employment (Croen et al., 2015; Lord et al., 2020). There is a strong genetic component (Tick et al., 2016) and evidence for epigenetic factors, including DNA methylation, Histone modification and chromatin remodelling, and dysregulation of micro RNAs, being implicated in autism aetiology (Waye & Cheng, 2018; Yoon et al., 2020). However, perinatal risk factors, such as gestational age (GA) may also contribute to the prevalence of autism through interaction with genetic predisposition or through independent environment mediated pathways (Hultman et al., 2002; Lee et al., 2022; Limperopoulos et al., 2008). This study will focus on the differences in prevalence and outcomes between preterm, full-term and post-term children and adolescents with and without autism.

Prematurity (birth before 37 weeks of GA) occurs in approximately 10% of children worldwide (WHO, 2012). It has repeatedly been linked to impaired child (neuro)development (Blencowe et al., 2013; Vohr, 2013), often with cognitive and/or psychological deficits persisting into adulthood and impairing quality of life and mental health (Howlin et al., 2004; Peralta-Carcelen et al., 2018; Rogers et al., 2018). Preterm children in the general population more often show a behavioural phenotype characterized by social, behavioural, and emotional difficulties alongside inattention/ hyperactivity, all features that resemble autism symptoms (Cassiano et al., 2016; Fitzallen et al., 2020; Johnson & Marlow, 2011). Individuals born preterm are at higher risk for childhood psychopathology in general (Burnett et al., 2011) and autism in particular (Allen et al., 2020; Atladottir et al., 2016; Fitzallen et al., 2020; Ng et al., 2017). This includes those born moderately to late preterm (MLPT, 34–36 weeks of GA) (Guy et al., 2015; Persson et al., 2020) who are often falsely thought to have similar outcomes as full-term children (Gill & Boyle, 2017; Johnson et al., 2015; Sanghera & Boyle, 2018). A recent meta-analysis reported an autism prevalence of 7% in preterm individuals (Agrawal et al., 2018) compared to around 1% in the general population (Lord et al., 2020). The prevalence of prematurity within an autism sample and the direct comparison to a matched control sample remains un-investigated.

Although the association between preterm birth and autism prevalence is well established, less is known about how preterm birth influences autism presentation. *Autistic*^1^ individuals born preterm are thought to show increased symptomology (Movsas & Paneth, 2012) and a distinct phenotypic expression of autistic traits and behaviours (Ure et al., 2016), with more deficits in social interaction and communication, but fewer restricted or stereotypical behaviours compared to full-term counterparts (Kalish et al., 2017).

To date, the limited number of studies comparing phenotypic characteristics between pre- and full-term born individuals with autism report contradicting results. Bowers et al. (2015) reported a greater percentage of sleep apnea, seizure disorders and attention deficit/hyperactivity disorder (ADHD) in autistic individuals born preterm (Bowers et al., 2015). Brayette et al. (2019) demonstrated significant modifications in cognitive abilities in individuals with autism born moderately to late preterm (MLPT) and early term (ET). However, contradicting Bowers et al. (2015) no differences were found with respect to behavioural assessment. The authors therefore concluded that the autism phenotype might be independent of term (Brayette et al., 2019). A comparison of pre- and full-term infants in a third study reported similar severity of behavioural autistic symptoms but differences in patterns of reciprocal social interaction (Chen et al., 2019). To conclude, previous studies show heterogeneous results concerning autism phenotype in individuals born preterm.

A neglected risk factor in autism is post-term birth (> 42 weeks of GA). More recent findings by Xie et al. (2017), as well as Persson et al. (2020), who report increased risk for autism in post-term children therefore highlight the necessity to investigate post-maturity and autism more closely. Aiming at assessing the relative risk for autism across the entire GA range Persson et al. (2020) used population-based data from national registries in Nordic countries. Although the risk was largest for very preterm individuals, it increased with deviation from 40 weeks of GA including MLPT and post-term individuals. Furthermore, individuals born post-term show long-term emotional problems and a higher risk for clinically relevant problem behaviour compared to full-term counterparts (El Marroun et al., 2012), thereby highlighting the need to investigate this population with regard to autism symptomology. To date only one study compared full- and post-term children with autism and reported a higher symptom severity in the latter (Movsas & Paneth, 2012). Similar to individuals born preterm birth, autistic children born post-term might also differ from their full-term counterparts in other aspects of autism presentation.

In conclusion, only few studies looked at the phenotypic expression of autism with regard to preterm birth with inconclusive results, and only one study considered the relevance of post-term birth. Samples of autistic individuals used were small and often recruited through a clinic, which might lead to bias in the form of higher comorbidity rates and problems (Berkson’s bias) (Berkson, 1946). To more closely investigate the association between GA and autism presentation, particularly in underrepresented post-term children, it is important to study autistic traits, social skills, and comorbid symptoms, which usually characterize the autism phenotype, in a population derived sample including various GA groups. Therefore, we aim to (1) determine the prevalence of pre- and post-term birth in autistic children and adolescents compared to a stratified and matched general population sample of children and adolescents to elucidate differences and (2) clarify inconsistencies regarding potential differences in the phenotypic expression of autistic traits, social skills, behaviours, and comorbid symptoms across GA groups. We hypothesize that the deviation from full-term birth together with a diagnosis of autism will additively lead to more severe presentation of autistic symptoms and comorbid problems in children and adolescents with autism born pre- and post-term. Identifying differences in autism phenotype between children of different GA groups, might help in focussing early prevention and intervention and allocate resources to the group most in need.

## Methods

### Sample

The current study used two different samples. A population derived sample of children and adolescents with autism and a sample of matched controls from the general population.

Data for the autism sample were obtained from the *Netherlands Autism Register* (NAR), a longitudinal cohort database, containing self- and parent-reported information on functioning and demographics in individuals with an autism diagnosis. Information in the NAR is provided by autistic people themselves and their families through annual online questionnaires. Questionnaire data for this study was mostly (97%) obtained through parents reporting on their autistic child (3% were provided by autistic individuals themselves). The NAR cohort has been published on widely (see https://www.nederlandsautismeregister.nl/english/). The current sample included 613 children and adolescents with a formal diagnosis of autism (mean age M = 14.01, SD = 3.63, range 3–24 years; mean age of diagnosis M = 5.63, SD = 2.53, based on *n* = 458 (77.58%)) received from a qualified clinician based on Diagnostic and Statistical Manual of Mental Disorders (4th ed.; DSM-IV) or Diagnostic and Statistical Manual of Mental Disorders (5th ed.; DSM-5). Parents/individuals have to confirm their autism diagnosis and disclose additional information on when, where, and by whom (psychiatrist/psychologist) the diagnosis was determined, as well as the measure used (ADOS or ADI-R) (Deserno et al., 2019). Moreover, official proof of autism diagnosis could be obtained for a subsample of individuals in the NAR. Patterns of functioning in this group were similar to the rest of the sample (Deserno et al., 2019). Additionally, previous findings on the validity of parent-reported autism diagnosis to a web-based register supports the validity of the register diagnosis (Daniels et al., 2012).

Within this original sample pregnancy length was reported by selecting “extremely premature” (< 28 weeks GA), “very premature” (28–32 weeks of GA), “premature” (33–36 weeks of GA), “on term” (37–41 weeks of GA) and “more than two weeks late” (> 42 weeks of GA). In the Netherlands GA is usually determined based on the first day of the last menstrual cycle plus 40 weeks. Six participants indicated “unknown” and were therefore excluded from analysis. Another person was excluded due to not providing date of birth. The final sample used in the analysis included 606 children and adolescents from the NAR with a mean age of 14.01 (SD = 3.63, range 3–24 age). Extremely premature, very premature and premature were grouped together resulting in a total of 70 children and adolescents born preterm, 451 born full-term, and 85 born post-term. Information on ethnicity was available for *N* = 571 individuals. Out of these 98% (*n* = 562) reported being Dutch. Parental education was medium to high for over 74% of the sample and the majority of the sample was Dutch. Further information on demographics in the three groups can be found in Table 1.

**Table 1:** Demographics of the autism sample

Characteristic	Full cohort (*n* = 606)	Preterm (*n* = 70)	Full-term (*n* = 451)	Post-term (*n* = 85)
Age^a^	14.01 (3.63)	13.43 (3.71)	13.99 (3.68)	14.6 (3.26)
Range	3–24	6–19	3–24	6–21
Sex^b^				
Male	469 (77.4)	60 (85.7)	340 (75.4)	69 (81.2)
Female	137 (22.6)	10 (14.3)	111 (24.6)	16 (18.8)
Birthweight^a^	3409.98 (665.25)	2308.26 (692.75)	3503.19 (486.40)	3820.05 (556.63)
Parental age at birth^a^				
Mother	31.88(4.42)	32.57 (4.10)	31.76 (4.35)	31.95 (5.02)
Father	34.52 (5.27)	34.75 (4.72)	34.52 (5.15)	34.31 (6.27)
Autism in the family^b^				
Yes	177 (29.2)	20 (28.6)	133 (29.5)	24 (28.2)
Education mother^b^				
Low	51 (8.4)	6 (8.6)	35 (7.8)	10 (11.8)
Medium	213 (35.1)	32 (45.7)	149 (33.0)	32 (37.6)
High	301 (49.7)	30 (42.9)	232 (51.4)	39 (45.9)
Unknown	41 (6.8)	2 (2.9)	35 (7.8)	4 (4.7)
Education father^b^				
Low	100 (16.5)	12 (17.1)	77 (17.1)	11 (12.9)
Medium	180 (29.7)	25 (35.7)	124 (27.5)	31 (36.5)
High	271 (44.7)	28 (40.0)	206 (45.7)	37 (43.5)
Unknown	55 (9.1)	5 (7.1)	44 (9.8)	6 (7.1)
Autistic traits^a^				
Age of diagnosis	5.62 (2.53)	5.49 (2.45)	5.65 (2.59)	5.60 (2.34)
AQ-short total score	81.75 (10.58)	79.41 (11.76)	81.65 (10.51)	83.85 (9.82)
SDQ total score	16.51 (6.03)	17.06 (5.53)	16.29 (6.05)	17.60 (6.33)

*SDQ* strength and difficulties questionnaire, *AQ-short* autism-spectrum-quotient-short

^a^Means are displayed with standard errors in parentheses

^b^Absolute number is displayed with percentage is in parentheses. Birthweight is presented in grams. Autism in the family asks for an autism diagnosis in other family members (son, daughter, father, mother, brother sister).

Matching on birth year, age of the mother at birth, sex and province in the Netherlands a representative general population comparison sample was randomly drawn from the Dutch birth register *Perined* with GA based on ascertained Dutch medical records, also calculating from the first day of the last menstrual cycle. More information can be found on https://www.perined.nl/s. The final comparison sample size was *N* = 593 individuals, as not all of the children and adolescents with autism could be matched.

### Measures

Aside from demographic questions participants completed the following:

*Autism-Spectrum-Quotient (AQ)-Short.* To assess parent-reported autistic traits, the 28-item AQ-short (Hoekstra et al., 2011) was used. Psychometric analysis identified fascination for numbers and patterns, and social behaviour as the two higher-order factors, with the latter including subscales for social skills, routine, switching and imagination. Responses for each question are indicated using a four-point Likert scale ranging from “1 = definitely agree” to “4 = definitely disagree”. The AQ-short total score shows an internal consistency between α = 0.77 and α = 0.86, and excellent test accuracy (specificity = 0.91; sensitivity = 0.94) with higher total scores representing higher symptom severity and impaired social skills.

*Strengths and difficulties questionnaire (SDQ).* Comorbid problems were measured in the form of comorbid symptoms such as emotional and behavioural problems with the widely used SDQ parent-report (Goodman, 1997, 2001). The SDQ consists of 25 items describing positive and negative attributes rated on a three-point Likert scale and shows satisfactory internal consistency (α = 0.73) and validity with high scores being linked to increased psychiatric risk. The five SDQ subscales assessing emotional symptoms, conduct problems, hyperactivity-inattention, peer problems and pro-social behaviour are measured with five items respectively. Scores on each subscale range from 0 to 10 with higher scores indicating more problems (except for the prosocial subscale). Factor structure and psychometric properties of the SDQ were found to be similar in a Dutch community sample of children and adolescents (Muris et al., 2003).

### Analyses

Prevalence rates of GA groups were compared between the autism sample and the matched general population sample using Pearson’s Chi-square test for homogeneity. Odds Ratios were calculated as a measure of effect size, indicating risk of autism diagnosis given pre- or post-term birth, using a binary logistic regression with two dummy variables. A multinomial logistic regression analysis was performed to compare autistic traits, patterns of symptoms and difficulties between individuals with autism across GA groups. AQ total score, SDQ total score and intelligence quotient (IQ) as a measure of cognitive functioning were used as predictors while controlling for sex and maternal age at birth of the child as potential confounders. Out of the subsample used for the logistic regression 85% reported their IQ based on a test result. To further validate the IQ scores, we calculated a correlation between parent-reported IQ and CITO (Central Institute for Test Development) scores, a standardized Dutch measure for educational achievement. This measure was found to be moderately to highly correlated with IQ in other studies (Bartels et al., 2002) and was significantly correlated with our parent-reported IQ scores, *r* = 0.33, *p* < 0.01.

## Results

Descriptive summary statistics of autism sample demographics can be found in Table 1.

### Prevalence of Gestational Age Groups and Risk of Autism Diagnosis

Frequencies for the three GA groups for both, the autism and the general population sample can be found in Table 2. Prevalence rates of GA groups differed between the autism and the general population sample (χ^2^ = 48.15, df = 2, *p* =  < 0.001). Compared to the general population, rates of preterm and post-term birth were elevated in autistic children and adolescents. Moreover, risk for autism was 1.8-fold higher among preterm-born individuals (*p* < 0.01, 95% CI = [1.22, 2.69]) and 4.5 times higher in post-term individuals (*p* < 0.001, 95% CI = [2.77, 7.32]) than in full-term counterparts.

**Table 2:** Frequencies of gestational ages by sample

	NAR (*N* = 606)	Perined (*N* = 593)
Gestational age (GA)		
< 37 weeks (preterm)	70 (11.56%)	45 (7.59%)
37–41 weeks (full- term)	451 (74.40%)	526 (88.70%)
> 42 weeks (post-term)	85 (14.03%)	22 (3.70%)

NAR = autism sample, Perined = matched representative general population sample

### Phenotypic Expression of Autistic Traits Across Different Gestational Age Groups

The results of the logistic regression analysis indicate, that when controlling for sex and maternal age at birth neither parent-reported IQ, nor AQ and SDQ total scores serve as predictors to differentiate across GA groups within a sample of children and adolescents with autism (see Table 3), thus there seems to be no association between measures of phenotypic expression of autism, comorbid symptoms, or IQ with GA. Consequently, preterm and post-term children and adolescents with autism do not seem to differ from full-term counterparts in their autistic traits, patterns of social interaction behaviour, comorbid symptoms and cognitive ability.

**Table 3:** Results from the multinomial logistic regression on phenotypic expression compared to full-term children

Gestational age group	Predictor	Chi square p-value	OR (95% CI)*
Full-term (*n* = 147)			
Preterm (*n* = 16)	AQ total score	.56	1.02 (.96, 1.07)
	SDQ total score	.21	1.07 (.96, 1.19)
	Cognitive ability (IQ)	.41	1.29 (.70, 2.38)
	Sex	.28	.31 (.04, 2.53)
	Maternal age at birth	.11	1.12 (.98, 1.28)
Post-term (*n* = 24)	AQ total	.39	1.02 (.98, 1.07)
	SDQ total score	.24	1.05 (.97–1.15)
	Cognitive ability (IQ)	.20	1.40 (.84–2.36)
	Sex	.49	.63 (.17, 2.33)
	Maternal age at birth	.35	1.06 (.94, 1.19)

Analysis was based on *N* = 187 autistic children and adolescents with full-term individuals as reference category.

*OR* odds ratio, *CI* confidence interval

## Discussion

This study aimed at highlighting the relevance of studying gestational age (GA) in relation to autism, clarifying current inconsistencies in findings and extending them to the post-term born population.

We compared prevalence rates of GA groups between a population-derived autism sample with clinical diagnosis and a matched sample of children and adolescents without autism from the general population. The prevalence of preterm birth was 1.5 times higher (11.56 in autism, 7.59 in comparison), and the prevalence for post-term birth was more than three times higher in autistic children and adolescents compared to the general population, with 14.03% of individuals in our autism sample being born after 42 weeks of gestation versus 3.7% in the comparison sample. These findings are likely an underestimation. Low socio-economic background has been linked with preterm delivery (Brumberg & Shah, 2015). Therefore, given the highly educated nature of our autism sample, the reported differences in prevalence rates, especially for preterm birth, are most likely an underestimation. Elevated prevalence rates of pre- and post-term birth within our autism sample together with the observed increased risk for autism diagnosis, especially in post-term individuals, stress the importance of investigating mechanisms underlying this association in the future. Moreover, they highlight the crucial need of including and separately investigating individuals born post-term who are also at risk of adverse developmental outcomes.

The present study did not find an association between GA and autistic traits, behavioural patterns and overall cognitive ability when controlling for sex and maternal age at birth, thereby supporting previous findings by Chen et al. (2019) and Brayette et al. (2019) in a larger sample of individuals with autism. Our results therefore indicate that there is no association between GA and autism phenotype and therefore no additive effect of prematurity on autistic traits and symptoms. Different explanations can be considered for our findings.

The first possible explanation for the lack of phenotypic difference between children and adolescents with autism born pre-, full- and post-term may be that autism as a pervasive developmental disorder impacts many domains of functioning and therefore masks the effects of GA. Once the disorder manifests in an individual’s behaviour, justifying a clinical diagnosis, GA at birth might not, as previously thought, have an additive impact on the presentation of autistic symptoms and traits. The overshadowing effect of autism would explain findings of differences in the general population regarding behaviours (Moster et al., 2008) and cognitive functioning (Ortiz-Mantilla et al., 2008) and the lack thereof in our study. Furthermore, symptoms characterizing the preterm behavioural phenotype in the general population might represent a subclinical form of autism that leaves more variance in various impairments.

Second, taking into account that children born preterm might receive more medical attention, identification of atypical behaviour might be facilitated in this group, elevating the possibility of early diagnosis. Being diagnosed early allows individuals to take part in early intervention programs and to access other services and support (Begeer et al., 2013) which has shown to be associated with better long-term outcomes (Lord et al., 2020).

Third, the children and adolescents in our autism sample were from an educated family background. Given results by Rosenberg et al. (2009) which show possible environmental modification of highly heritable traits, it is also plausible that environmental factors might have a beneficial impact on the severity of autistic traits. The favourable effect of a highly educated, supportive environment positively influencing development might be especially strong for preterm children (Belsky et al., 2007) due to their vulnerability to their environment (Shah et al., 2013), as well as high plasticity and variability in outcomes (Wolke, 2018).

Post-term birth was a risk factor for autism diagnosis in our study. However, it was not associated with a different phenotypic expression of autism in our study although a higher autism severity (Movsas & Paneth, 2012), more behavioural and emotional problems (El Marroun et al., 2012) and a higher rate of neurodevelopmental disorders (Katarina Lindström et al., 2005) were previously reported in this population. Contrary to this, Gardener and colleagues (2011) argue that post-term birth is in fact a strong protective factor with regard to the risk of developing autism, therefore also buffering adverse effects. There is a lot of uncertainty regarding post-term birth and its association with autism and related outcomes. Future research in the field of autism symptomology and comorbid (mental) health problems in association with GA should therefore more closely look at outcomes for post-term individuals.

Our study specifically focused on GA and autism (phenotype). However, there are other factors that are associated with an increased risk for preterm delivery and/or autism itself, concerning characteristics of the mother, the pregnancy and delivery, as well as infant characteristics. Characteristics of the mother include factors such as maternal anaemia (Lone et al., 2004) and maternal ethnicity (Hultman et al., 2002; Ng et al., 2017) that are associated with increased risk for premature delivery and autism respectively. Several factors implicated in autism risk can be subsumed under the umbrella of antenatal care, pregnancy and delivery. These include among others hypertensive diseases, chorioamnitis and hemorrhage during pregnancy (Gardener et al., 2011; Limperopoulos et al., 2008), as well as clustering of pregnancy complications (Ng et al., 2017), caesarean delivery and multiple births (Gardener et al., 2011; Hultman et al., 2002). Furthermore, characteristics of the infant such as low birth weight (Kolevzon et al., 2007) and intrauterine growth restriction or small-for-gestational-age status (Gardener et al., 2011; Lampi et al., 2012; Ng et al., 2017), especially in premature infants (Moore et al., 2012), are associated with autism. Besides environmental factors genetic disorders such as Sotos syndrome (Lane et al., 2017) or sex chromosome aneuploidy (Tartaglia et al., 2017) were also associated with a higher likelihood of autism. Prospective studies investigating etiological pathways including all of the above-mentioned factors and focusing on critical time points of vulnerability can be helpful to better understand how these factors are associated with autism development and presentation. We did not consider information on medical comorbidity among autistic individuals. Medical conditions are common among autistic individuals (Croen et al., 2015) and future research might benefit from including medical conditions in the description of autism phenotype.

## Limitations

Several limitations should be considered when interpreting the findings of this study. A major limitation is the cross-sectional nature of the data that does not allow for causal conclusions. Moreover, the data in this study are acquired via parent report, including autism diagnosis. Previous findings in an online register could verify parent reported diagnosis and point towards their validity (Daniels et al., 2012), however misclassification of individuals could have a significant influence on the results. Furthermore, although parent reported questionnaire data can provide valuable insights into subjective difficulties noted by autistic children’s and adolescent’s caregivers, we cannot rule out imprecision of the reported data and uncertainty about the generalizability of findings. The use of the SDQ allowed us to look at symptoms of comorbid conditions on a subthreshold level rather than a diagnosis as binary outcome. However, due to the later inclusion of the SDQ in the NAR survey, information on the measure was not available for all individuals. This limited our power to detect differences in comorbid symptoms among our sample of autistic individuals. Our sample included individuals diagnosed under DSM-IV or DSM-5 criteria, which differ especially in the inclusion of Asperger syndrome in the broader autism spectrum and allowing for comorbidity with ADHD. This might have introduced heterogeneity to our sample, as some individuals diagnosed under DSM-5 would not be diagnosed under DSM-IV criteria. This could have influenced the presentation of autism symptoms and comorbid problems observed in this sample. Furthermore, the majority of our sample stems from an educated background. This highly selected nature of the sample might have reduced the amount of variance in autistic traits and comorbid symptoms and have influenced the results*.* Children and adolescents from families with lower social, educational, and socio-economic background might show more problems (Karolina Lindström et al., 2009; Potijk et al., 2013). Future studies should look more closely at these influencing factors which can increase or decrease risk for adverse development itself (Potijk et al., 2013). Autistic children and adolescents in our sample were mostly born moderate to late preterm with only few children born extremely preterm. Although previous research points towards increased risk for adverse developmental outcomes in this group (Johnson et al., 2015), including a greater percentage of children born extremely or very preterm to cover the whole range of prematurity might foster new insights on how GA influences development and presentation of autism.

## Conclusion

The prevalence of post-term birth in the autism sample was highly elevated, and across samples post-term children had a particularly high risk for autism. The higher prevalence rates of preterm and post-term birth in an autism sample compared to the general population highlight the need for further investigating the link between GA, autism, and associated outcomes. Overall, it seems hard to disentangle the effects of autism and GA on behavioural outcomes. Although, our results point towards no differences in the autism phenotype between pre-, full- and post-term children, the possibility of distinct phenotypes cannot be completely ruled out. Therefore, future research should aim at clarifying whether there are significant differences in autistic traits between different GA groups and determine their underlying mechanisms, for example by also taking into account genetic data. Most importantly, the findings of the current study call for the highly neglected population of individuals born post-term to be included into research questions regarding GA and developmental trajectories, and various psychiatric and neurodevelopmental outcomes, including autism. Identifying challenges and outcomes faced by the post-term population throughout their development will help to establish early support and intervention for these individuals and their families similar to preterm counterparts.

## Footnotes

1 We use a mix of identity-first language (e.g. autistic individual) and person-first language (e.g. individual with autism) in this paper in accordance with preferences reported by researchers, autistic people and their families (Kenny et al., 2016).
